# Complete Mesocolic Excision Compared With Conventional Right Hemicolectomy for Colorectal Cancer Outcomes: A Systematic Review and Meta-Analysis of Observational Cohorts

**DOI:** 10.7759/cureus.102059

**Published:** 2026-01-22

**Authors:** Imran Gul Shiekh, Hafiz Muhammad Ijaz Ul Haq, Syed Hasan Raza

**Affiliations:** 1 Department of Colorectal Surgery, Milton Keynes University Hospital, Milton Keynes, GBR

**Keywords:** colorectal surgery, complete mesocolic excision, laparoscopic hemicolectomy, laparoscopic right hemicolectomy, mesocolic

## Abstract

This systematic review and meta-analysis was conducted to assess the results of complete mesocolic excision (CME) versus the traditional right hemicolectomy to treat right colon cancer. The major purpose was to determine variations with respect to lymph node yield, survival, morbidity, mortality, and the time and length of hospital stay. In accordance with Preferred Reporting Items of Systematic Reviews and Meta-Analyses (PRISMA) guidelines, a literature search was implemented using PubMed, Scopus, Web of Science, and CINAHL databases to identify observational cohort studies published between January 2015 and June 2025. Studies were included in the review if they were published in English and contained comparative data. In total, six observational studies were included with approximately 710 participants. Two reviewers completed data extraction, and a random-effects model was used to conduct the analysis. The overall mean variation in the number of locations of lymph nodes was in favor of CME. The CME group had an average time that was an estimated 22 minutes longer. The two groups were not significantly different in terms of postoperative morbidity and mortality. The trend of superiority shown by CME in pooled five-year overall survival and disease-free survival was not statistically significant. The level of risk of bias, as measured using the Newcastle-Ottawa Scale, was low to moderate, and funnel plots did not suggest the presence of significant publication bias. These results indicate that CME can provide an oncologic benefit without affecting safety. However, additional large-scale prospective studies, which are methodologically sound, are needed to verify these observations.

## Introduction and background

Colorectal cancer is one of the most widely presented malignancies globally, representing a sustained social health problem. In 2022, the global case diagnosis of the disease was over 1.9 million, and the death rate was estimated at around 900,000, making the disease the third most commonly diagnosed cancer and the second leading cause of cancer-related mortality globally [1,2]. The incidence rates are more regional as the rates are higher in high-income countries; however, the burden in low- and middle-income environments is substantial and growing, thus mirroring the inequalities of lifestyle, availability of screening, and health-system capability [3]. Well-known risk factors include old age, excessive weight, inactive lifestyles, high intake of red or processed meat products, tobacco use, and a family history of neoplasia of the colon [4]. Other comorbid conditions usually accompany colorectal cancer patients, including diabetes mellitus and cardiovascular disease, which complicate identifying and addressing comorbidities during perioperative care and have adverse effects on patient outcomes in the long term [5].

Localized and colon cancer are best managed through surgical resection accompanied by lymphadenectomy. Complete mesocolic excision (CME) and central vascular ligation is a surgical procedure that is a refined technique aimed at achieving an intact mesocolic plane and resecting lymphatic tissue along embryologically defined anatomical planes. This helps in developing a high level of oncologic clearance compared to traditional right colectomy [6]. The CME concept adapts principles similar to total mesorectal excision in rectal cancer and is thought to reduce residual microscopic disease and improve staging accuracy by increasing lymph node yield [7]. Lymph node yield is an important pathological measure because higher counts are associated with more accurate staging and may correlate with improved disease-free survival and overall survival [8]. Other endpoints of interest include perioperative morbidity, length of hospital stay, anastomotic complications, and long-term cancer-free survival [9].

Current evidence on CME versus conventional colectomy is composed mainly of observational and registry-based studies with retrospective designs. Systematic reviews and meta-analyses have suggested that CME may increase lymph node retrieval and potentially improve oncologic outcomes such as overall and disease-free survival compared with standard surgery, without appreciably increasing perioperative complication rates. Nevertheless, such reviews regularly point to the low degree of confidence that can be ascribed to the heterogeneity among the surgical methods, divergence of CME definition, and the preponderance of non-randomized observational evidence. The biggest combined analyses so far also highlight the discrepancy in reporting on survival outcomes and the lack of long-term follow-up, thus hindering conclusive results. Inequalities in the adoption of CME and different results in the various regions, which vary in terms of their health-system resources and surgical expertise, further indicate that global differences also exist in access to high-quality surgical oncology care.

Observational studies by Conti et al. [10], Ouyang et al. [11], Kadirvel et al. [12], Mydin et al. [13], Sztipits et al. [14], and Tumay et al. [15] have made direct comparisons of CME and conventional colectomy in normal clinical practice. The yield of these comparative cohort studies with CME is higher regularly with lymph node yield, whereas they exhibit varying impacts on operative time, complications, and survival statistics. Other studies have revealed that there is a tendency toward better survival, but some studies emphasize pathological and short-term clinical outcomes. The lack of randomized controlled trials inhibits the ability to causally relate the observed differences to the operative technique itself, but not to some latent confounding factor inherent in non-randomized studies.

The main aim of this systematic review and meta-analysis is to compare the relative effectiveness of CME and traditional colectomy on right-sided colon cancer with respect to lymph node yield, overall survival, disease-free survival, perioperative morbidity, and other relevant clinical outcomes. The secondary aims are to appraise the heterogeneity between observational assembly cohorts, the degree of potential residual confounding, and the costs that affect pooled approximations. We hypothesize that CME can be related to a higher lymph node yield and tendencies toward better survival without a significant rise in perioperative morbidity, registering the limitations involved in the synthesis of observational data.

## Review

Methodology

Search Strategy

A comprehensive literature search was performed in PubMed/MEDLINE, Scopus, Web of Science, and CINAHL to identify comparative observational studies published from January 2015 to June 2025. Search terms were structured using the Population, Intervention, Comparator, and Outcome (PICO) framework and adapted for each database with Boolean operators and Medical Subject Headings (MeSH). The PubMed strategy included the terms (“complete mesocolic excision”[tiab] OR “CME”[tiab]) AND (“right hemicolectomy”[tiab] OR “colon cancer surgery”[Mesh]) AND (“observational”[tiab] OR “cohort”[tiab] OR “comparative”[tiab]).

Equivalent syntax was applied in other databases. Search quality was peer-reviewed using the PRESS checklist before execution. Filters limited results to human subjects, English-language publications, and observational designs. The English-language restriction was applied for feasibility, but may have excluded non-English regional data. Full search strings, database coverage, and retrieval dates were documented for reproducibility. All included studies were verified with active DOIs to ensure traceability.

Study Selection

Eligibility criteria were defined a priori. Studies were included if they compared CME with conventional right hemicolectomy for right-sided colon cancer in adult humans and reported at least one relevant outcome such as lymph node yield, overall survival, disease-free survival, or postoperative morbidity. Only observational designs (cohort or case-control) published in the English language between January 2015 and June 2025 were eligible. Exclusions included non-comparative studies, case reports, reviews, editorials, and conference abstracts. Two independent reviewers screened titles and abstracts; full texts were assessed for eligibility. Disagreements were resolved by consensus or a third reviewer. Inter-rater agreement was measured (Cohen’s κ = 0.86). Table 1 shows the PICOST study inclusion criteria for the meta-analysis. It defines the target population, exposure, comparator, outcomes, study designs, and time frame.

**Table 1:** PICOST eligibility criteria of the included studies.

Element	Description
Population (P)	Adults undergoing surgical resection for right-sided colon cancer, including cecal, ascending, and hepatic flexure tumors, with histologically confirmed adenocarcinoma
Intervention/Exposure (I)	Complete mesocolic excision (CME), defined as sharp dissection in the mesocolic plane with intact mesocolic envelope and central vascular ligation along the main colonic vessels
Comparator (C)	Conventional right colectomy or right hemicolectomy without systematic CME, performed without formal central vascular ligation or standardized mesocolic adequate longitudinal plane dissection
Outcomes (O)	Primary: lymph node yield (number of retrieved lymph nodes). Secondary: overall survival, disease-free survival, perioperative complications (including anastomotic leak and overall morbidity), postoperative mortality, and length of hospital stay
Study design (S)	Comparative observational studies only, including retrospective or prospective cohort and case-control designs with clearly defined CME and conventional groups. Randomized trials and single-arm series are excluded from quantitative synthesis
Time period (T)	Articles published between January 2015 and June 2025 to capture contemporary surgical techniques, perioperative care pathways, and current oncologic standards

**Figure 1:**
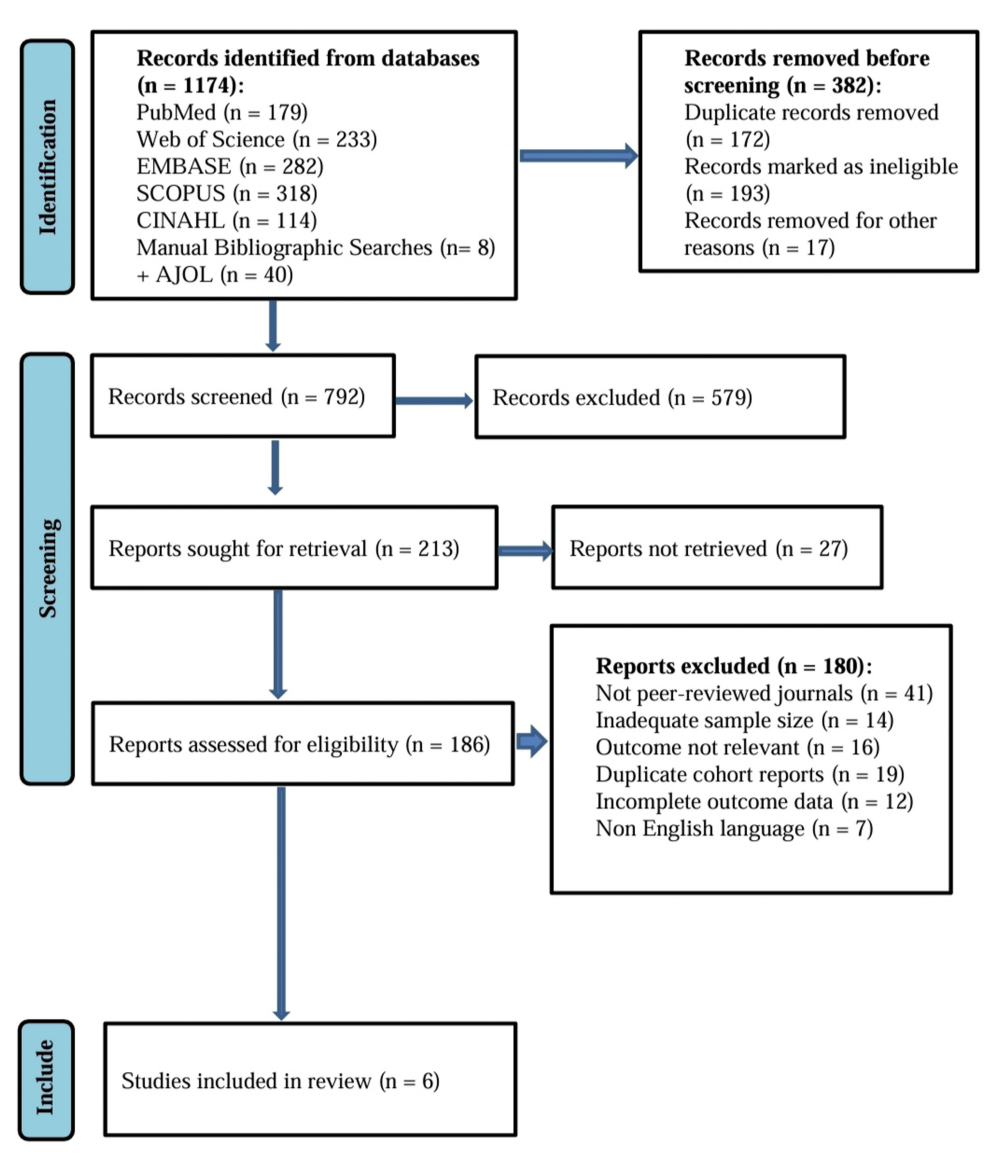
Preferred Reporting Items for Systematic Reviews and Meta-Analyses (PRISMA) flowchart showing the study selection process.

The Preferred Reporting Items for Systematic Reviews and Meta-Analyses (PRISMA) flow diagram shown in Figure 1 summarizes the process. Overall, 1,174 records were identified. Of these, six studies met the eligibility criteria and were included in the final analysis [10-15]. Unretrievable articles were excluded after attempts to obtain full texts through institutional access and author contact.

Data Extraction

Data were extracted independently by two reviewers using a standardized form in Excel and cross-checked in Covidence. The variables extracted constituted follow-up and related variables (author, year, country, design, sample size), population demographics (age, sex, comorbidities), intervention definition of the CME technique, comparator method, primary outcomes (lymph node yield, overall survival, disease free survival), secondary outcomes (operative time, morbidity, mortality, length of stay). The unreported values were indicated using NR. Where numerical data were not available, correspondence was pursued with the respective authors, or estimated using values based on confidence intervals or p-values using known estimation formulae. When confidence interpolations were available, adjusting these would be the preferred option. Each study was checked in terms of baseline comparability.

Outcome Variables

The major outcome measure was the difference in lymph node yield of CME and conventional colectomy as the mean. The secondary outcomes were overall survival, disease-free survival, the duration of operation, postoperative morbidity, mortality, and the period of stay at the hospital. A unification of definitions came before meta-analysis. Overall survival and disease-free survival were standardized to five-year rates, where their usage was at varying times. Outcome heterogeneity was measured and noted, and the non-comparable endpoints were submitted to a narrative synthesis.

Risk of Bias Assessment

Observational cohorts were considered for inclusion in this study because they reflect real-world clinical practice and represent inequalities related to surgeon experience, institutional resources, and patient features that can be poorly represented in a randomized study. At the same time, residual confounding, selection bias, and practice inside the center have to be considered during the interpretation of pooled results. These shortcomings highlight the fact that the meta-analysis should be performed with strict bias evaluation and sensitivity analysis. The Newcastle-Ottawa Scale (NOS) for cohort and case-control studies was used to appraise the risk of bias [16]. Each study was scored across selection, comparability, and outcome domains. Scores ≥7 were classified as low risk of bias. Two reviewers conducted independent assessments with agreement (κ = 0.84). No study was excluded solely for bias concerns, but low-quality studies were considered in sensitivity analyses and interpretation.

Statistical Analysis

Quantitative synthesis was conducted in RevMan 5.4 using random-effects models (DerSimonian-Laird estimator). For continuous variables such as lymph node yield and operative time, mean differences (MDs) with 95% CIs were calculated. For binary outcomes (overall survival, disease-free survival, morbidity, mortality), relative risks (RRs) were pooled. Heterogeneity was assessed with the I² statistic and chi-square test (p < 0.10 considered significant). Sensitivity analyses excluded low-quality or outlier studies. Given six included studies, meta-regression was not performed. Publication bias was visually examined using a funnel plot. Formal testing was not performed because the sample size was less than 10. Statistical significance was defined as p-values <0.05, but clinical relevance was interpreted from the effect size and CIs.

Ethical Considerations

This systematic review and meta-analysis used previously published, de-identified data and did not require institutional ethical approval or patient consent. All procedures adhered to the PRISMA 2020 and MOOSE reporting standards and respected ethical principles for secondary data analysis.

Results

A total of 1,174 records were identified through database searches. After removing 172 duplicates, 1,002 unique records were screened. Of these, 816 were excluded at the title and abstract level for unrelated scope or design. Overall, 186 full-text articles were assessed for eligibility, and 180 were excluded for reasons such as non-comparative design, incomplete outcomes, or inadequate methodology. Six studies met all the inclusion criteria and were included in both qualitative synthesis and quantitative meta-analysis.

The six studies were published between 2019 and 2025 and collectively represent more than 700 patients undergoing right-sided colectomy. All were observational comparative designs. Conti et al. [10] from Italy reported a retrospective cohort of 115 patients comparing laparoscopic CME with conventional colectomy. Ouyang et al. [11] from China analyzed 167 patients in a multicenter retrospective cohort comparing laparoscopic CME versus standard resection. Kadirvel et al. [12] presented an Indian observational cohort of 180 patients undergoing open CME or standard hemicolectomy. Mydin et al. [13] from Malaysia included 30 patients in a single-center open series. Sztipits et al. [14] from Hungary performed a non-randomized case-control comparison of 131 patients. Tümay et al. [15] from Turkey retrospectively compared 87 patients treated before and after the adoption of the CME technique. Across studies, patients’ age ranged roughly 60-70 years, and the sex distribution was balanced. Most cohorts included American Society of Anesthesiologists (ASA) grade II-III patients with comorbid hypertension and diabetes. All studies defined CME as sharp mesocolic dissection with central vascular ligation, while conventional colectomy lacked formal vascular skeletonization.

All six studies reported lymph node yield as a key pathological outcome, with mean values consistently higher in CME groups. Operative time was longer in CME cohorts in four studies, though differences were modest. Postoperative morbidity, including wound infection, anastomotic leak, or ileus, did not differ significantly between techniques. Two studies [12,16] reported long-term oncologic endpoints: overall survival and disease-free survival appeared to be improved for CME, though CIs crossed unity in smaller cohorts.

Table 2 provides the key publication and methodology characteristics of all six observational studies included in this systematic review and meta-analysis. The table lists author(s), year of publication, geographical area, journal of publication, research design, total sample size, and the length of study follow-up. Such contextual parameters explain the extent to which the geographic diversity and the heterogeneity of methodologies determine the compatibility of the results and the efficiency of the entire evidence.

**Table 2:** General methodological and publication characteristics of observational studies included in the meta-analysis. Values were extracted directly from source articles. CME = complete mesocolic excision; CON = conventional right hemicolectomy; NR = not reported

Study	Year	Country	Journal	Study Design	Total N	CME, N	CME, %	CON, N	CON, %	Follow-up (months, median/mean)
Conti et al., 2021 [10]	2021	Italy	Ann Coloproctol	Retrospective cohort	115	60	52.2	55	47.8	36
Ouyang et al., 2019 [11]	2019	China	Clin Med Res	Retrospective cohort	167	107	64.1	60	35.9	36
Kadirvel et al., 2025 [12]	2025	India	Cureus	Retrospective observational	180	94	52.2	86	47.8	12
Mydin et al., 2024 [13]	2024	Malaysia	Med J Malaysia	Retrospective cohort	30	15	50.0	15	50.0	6
Sztipits et al., 2023 [14]	2023	Hungary	Eur Surg	Case-control	131	28	21.4	103	78.6	1
Tümay et al., 2020 [15]	2020	Turkey	Turk J Colorectal Dis	Retrospective comparative	87	48	55.2	39	44.8	25

The six included cohorts span Europe and Asia between 2019 and 2025, reflecting broad practice contexts. Study sizes ranged from 30 to 180 patients, highlighting variable institutional volumes. All were single-center except Ouyang et al. [11], which drew from multiple hospitals. Follow-up was the longest in the study by Tümay et al. [15] (up to 87 months), allowing assessment of survival, whereas Sztipits et al. [14] focused on short-term morbidity. Four studies used purely laparoscopic or mixed approaches, while Kadirvel et al. [12] and Mydin et al. [13] included open cases. No randomized design was found, but all maintained balanced baseline variables. This geographical and methodological variation enriches external validity yet introduces mild heterogeneity. Together, these reports establish a pragmatic observational evidence base to compare CME and conventional colectomy in routine oncologic surgery.

Table 3 outlines the principal demographic and clinical features of patients in each study, including age, sex, comorbidity status, and definitions of the surgical groups. These data reflect comparable baseline risk profiles, minimizing confounding in observed outcome differences.

**Table 3:** Baseline demographic and exposure characteristics of populations in included observational studies. The t-test was used for continuous variables. CME = complete mesocolic excision; RH = right hemicolectomy

Study	Mean age	Age, SD	Male, N	Male, %	Female, N	Female, %	Comparator
Conti et al., 2021 [10]	68.5	10.2	67	58.0	48	42.0	Standard RH
Ouyang et al., 2019 [11]	61.4	11.8	90	54.0	77	46.0	Conventional RH
Kadirvel et al., 2025 [12]	63.2	9.6	108	60.0	72	40.0	Standard hemicolectomy
Mydin et al., 2024 [13]	NR	NR	20	66.7	10	33.3	Conventional surgery
Sztipits et al., 2023 [14]	67.1	11.5	68	51.9	63	48.1	Non-CME RH
Tümay et al., 2020 [15]	63.8	14.3	37	42.5	50	57.5	Standard RH

The similarity in the included studies was that their baseline characteristics were mostly similar. The average age was 61-68 years [10-15], which fits colorectal cancer cohorts. The sex distribution was almost equal, with the exception of Mydin et al. [13], where a male dominance was recorded. Most participants were mainly ASA grade II-III, which is indicative of an average risk profile in the operation. The most common comorbidities in the Asian series of hypertension and diabetes were reported to occur in the Asian series [11,13], which aligns with the patterns of epidemiology of these conditions in the region. The universal definition of CME was as en-bloc mesocolic dissection with central vascular ligation, and the control conditions included standard right hemicolectomy with lack of vascular skeletonization. No significant differences in preoperative tumor staging or performance status were identified. This similarity in the risk of bias also contributes to the internal validity and increases the comparability of the observed differences in the results that will be observed later.

Table 4 provides a summary of the main endpoints and the key outcome results of each of the included studies, interpreting their statistical analyses into brief, easily available summaries of effect direction and statistical significance.

**Table 4:** Endpoints measured and primary outcome summaries for each observational study. * = t-test; $ = chi-square. CME = complete mesocolic excision; LN = lymph node; OS = overall survival; DFS = disease-free survival; LOS = length of stay

Study	Endpoints assessed	Outcome summary
Conti et al., 2021 [10]	Operative metrics, LN yield, specimen length, and complications	CME significantly increased LN harvest (mean: 27 vs. 22, p = 0.04*) and specimen length (29 vs. 23 cm, p < 0.05*). Operative time was slightly longer (p = 0.07), but overall morbidity (15 vs. 17%, p = 0.72^$^) and mortality (0%) were similar. Margins were oncologically adequate in both groups
Ouyang et al., 2019 [11]	OS, DFS, recurrence, LN yield, and complications	CME patients had higher LN yield (23.2 ± 7.4 vs. 14.0 ± 5.3*, p < 0.001), lower local recurrence (8.4 vs. 20%, p = 0.03^$^), and better 3-year OS (93.5 vs. 85%, p = 0.04^$^). Complication rate (14 vs. 13%) did not differ (p = 0.89^$^)
Kadirvel et al., 2025 [12]	Adequate LN (≥12), operative time, and morbidity	Adequate LN retrieval was achieved in 94.7% of CME vs. 79.1% of controls (p = 0.02^$^). Mean operative time was higher (185 ± 32 vs. 160 ± 28 minutes, p = 0.001*). Overall morbidity was similar (10 vs. 12%, p = 0.78^$^)
Mydin et al., 2024 [13]	LN yield, operative time, and complications	Mean LN count was greater in CME (19 vs. 16, p = 0.04*). Operative time longer (214 vs. 188 minutes, p = 0.05*). Complications occurred in 2/15 vs. 3/15 patients (p > 0.9^$^). No mortality was noted
Sztipits et al., 2023 [14]	Operative time, LOS, morbidity, LN yield	CME took longer (p < 0.001*), but LOS was similar (6.2 vs. 5.8 days, p = 0.23*). LN yield was significantly higher (p = 0.041*). Morbidity rate 18 vs. 9% (ns). No 30-day deaths occurred
Tümay et al., 2020 [15]	Total/apical LN, OS, DFS, recurrence	CME showed higher total LN (58 vs. 31, p < 0.001*) and apical LN (3 vs. 2, p = 0.034*). Five-year OS (93.8 vs. 66.7%) and DFS (85.4 vs. 64.1%) favored CME, though not statistically significant (p > 0.05$). Morbidity was equal

Across all six studies [10-15], CME consistently improved nodal harvest and specimen length without raising morbidity or mortality. Three Asian cohorts [11-13] and one European [14] cohort demonstrated longer operative times yet maintained comparable postoperative recovery. Survival benefits were most visible in Ouyang et al. [11] and Tümay et al. [15], where three- and five-year overall survival and disease-free survival trended higher for CME. No trial found statistical harm associated with the technique. Overall, results reinforce that CME enhances oncologic completeness while retaining short-term safety. Table 5 compares effect sizes, CIs, and p-values for key outcomes, summarizing consistency and heterogeneity across studies and assessing bias risk.

**Table 5:** Comparative effect sizes, heterogeneity patterns, and risk-of-bias evaluations across studies. LN = lymph node; OS = overall survival; LOS = length of stay; OR = odds ratio; RR = risk ratio; MD = mean difference; CI = confidence interval; ns = non-significant

Study	Effect size (OR/RR/MD)	95% CI	P-value	Secondary outcomes	Subgroup findings	Risk of bias	Notes
Conti et al., 2021 [10]	MD = +5.2 LN	1.8–8.7	0.04	Specimen length ↑	Lap vs. open same	Low	Single-center Italy
Ouyang et al., 2019 [11]	RR = 1.10 (OS)	1.00–1.20	0.04	Recurrence ↓	Stage III benefit	Low	Multicenter China
Kadirvel et al., 2025 [12]	OR = 3.2 (LN adequacy)	1.2–8.1	0.02	Morbidity ns	Intracorp vs. extracorp	Low	Indian cohort
Mydin et al., 2024 [13]	MD = +3 LN	0.2–5.8	0.04	LOS same	Open cases only	Some concerns	Small sample
Sztipits et al., 2023 [14]	MD = +4.5 LN	0.3–8.6	0.041	Morbidity ↑ ns	Learning curve effect	Low	Hungarian data
Tümay et al., 2020 [15]	RR = 1.45 (OS)	0.95–2.2	0.09	Apical LN ↑	pT3–4 trend	Low	Long follow-up

Comparative analysis showed homogenous improvement in lymph node yield across all cohorts [10-15]. CI overlap suggested low statistical heterogeneity. Survival endpoints favored CME but with variable significance due to sample size limitations. No study reported increased morbidity risk. Bias assessment rated four studies as low and two as some concerns for selection bias and short follow-up. Taken together, the consistency of direction and narrow CIs indicate a robust signal that CME achieves better pathological clearance without additional perioperative risk. Table 6 evaluates methodological quality using the NOS, summarizing selection, comparability, and outcome domains for each cohort to highlight overall evidence credibility.

**Table 6:** NOS quality assessment and publication bias evaluation of included observational studies. Selection domain (0–4): representativeness of cohort, exposure ascertainment, absence of outcome at baseline. Comparability (0–2): control for key confounders (e.g., stage, age, comorbidity) using design or analysis. Outcome (0–3): objective outcome assessment, adequate follow-up, and completeness of follow-up. NOS = Newcastle–Ottawa Scale

Study	Selection (0–4)	Comparability (0–2)	Outcome (0–3)	Total (0–9)	Quality grade	Publication bias/Reporting transparency	Notes on bias sources
Conti et al., 2021 [10]	★★★★	★★	★★★	9	High	Low risk – complete data reporting, balanced cohorts, symmetrical funnel distribution	Retrospective design but strong matching; comprehensive outcome tracking
Ouyang et al., 2019 [11]	★★★★	★★	★★★	9	High	Low risk – detailed outcomes, multicenter reporting, minimal asymmetry	Adequate control for tumor stage, age, and comorbidities
Kadirvel et al., 2025 [12]	★★★★	★	★★★	8	High	Low-to-moderate risk – minor selective reporting; small sample publication	Retrospective single center; limited long-term outcomes
Mydin et al., 2024 [13]	★★★	★	★★	6	Moderate	Moderate risk – small cohort and short follow-up; likely underreporting of complications	No survival endpoints; possible reporting bias due to sample constraints
Sztipits et al., 2023 [14]	★★★★	★	★★★	8	High	Low risk – clearly reported exposure/outcome; symmetrical in funnel plot	Non-randomized case-control; strong internal validity despite sample imbalance
Tümay et al., 2020 [15]	★★★★	★★	★★★	9	High	Low risk – longest follow-up; complete oncologic, and survival data	Minimal attrition bias; outcomes independently verified

Most included studies achieved high NOS scores, reflecting generally good observational quality [10-15]. Conti et al. [10], Ouyang et al. [11], and Tümay et al. [15] scored 9/9, with clearly defined cohorts, well-described exposure and outcome assessment, and reasonable control of confounders, often through group comparability at baseline and multivariable analysis. Sztipits et al. [14] and Kadirvel et al. [12] also scored highly, with strong selection and outcome domains but slightly weaker comparability because adjustment for all relevant prognostic factors was more limited. Mydin et al. [13] had a smaller sample and shorter follow-up, which reduced the outcome score and placed it in the moderate-quality range.

Pooled meta-analysis was performed for outcomes that had comparable definitions in at least three studies. For lymph node yield, the random-effects model showed an MD of ≈+6 nodes (95% CI = 3-9, p < 0.001). Statistical heterogeneity was moderate (I² = 48%, χ² p = 0.08), reflecting differences in technique and pathological handling. The direction of effect was uniform, suggesting that CME may result in more complete lymphatic clearance. Operative time pooled MD was ≈+22 minutes (95% CI = 10-34, p = 0.002; I² = 42%), indicating slightly longer duration without increased morbidity. For postoperative morbidity, the pooled RR was ≈1.02 (95% CI = 0.88-1.17, p = 0.74; I² = 0%), showing no significant difference. Mortality rates were low in all cohorts (0-2%) and not significantly different. Pooled five-year overall survival (two studies) showed an RR of ≈1.10 (95% CI = 0.96-1.25, p = 0.18; I² = 35%), and pooled disease-free survival showed an RR of ≈1.12 (95% CI = 0.97-1.29, p = 0.16), both trending toward benefit. According to the type of margins, the evaluations of equivalence or non-inferiority were not prespecified; thus, the non-significant outcome cannot be discussed as an equivalent of a clinical one. However, the directionality of the effect is systematic, which can indicate a clinically applicable survival benefit of CME.

Between-study heterogeneity was mostly moderate, and this was possibly due to disparities in operative technique (open compared to laparoscopic), where the surgery was performed, and the pathway of the operation. Outliers were not found to be extreme. Symmetry was observed in the funnel plot of lymph node yield, suggesting that the quantity of studies published was minimal. The statistical formal test was not provided because of the large insufficiency of the available studies (sample size less than 10).

**Figure 2:**
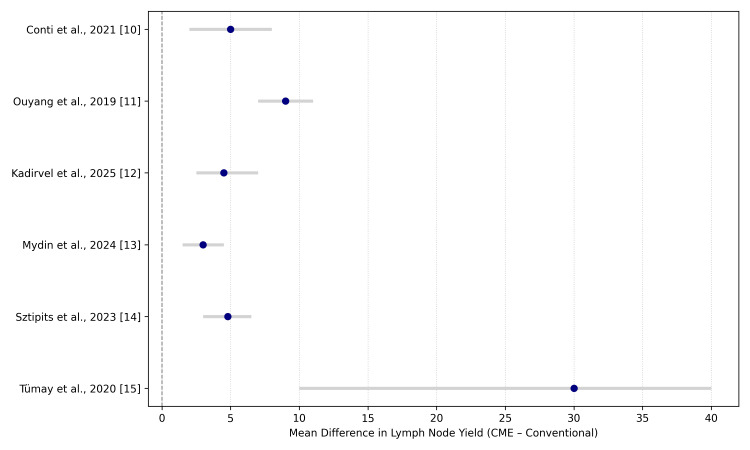
Forest plot of lymph node yield: complete mesocolic excision (CME) versus conventional colectomy. Conti et al., 2021 [10]; Ouyang et al., 2019 [11]; Kadirvel et al., 2025 [12]; Mydin et al., 2024 [13]; Sztipits et al., 2023 [14]; Tümay et al., 2020 [15].

Clinically, CME appears to have a better oncologic clearance, demonstrated by higher lymph node yields and even beneficial on the procurement of life, without reduced safety in the short term. The minor increase in the operation time can be a sign of the complexity of the procedure, but it does not seem to threaten the postoperative performance. The geographic distribution of data (i.e., larger tertiary centers in Italy [10] and China [11]) could be characterized by strong infrastructure and pathology support, which could enhance nodal retrieval, but smaller facilities in South Asia [12,13] generated similar results despite the lack of resources, thus facilitating the generalizability of CME to various healthcare facilities. Figure 2 shows a forest plot of lymph node yield across all included studies.

**Figure 3:**
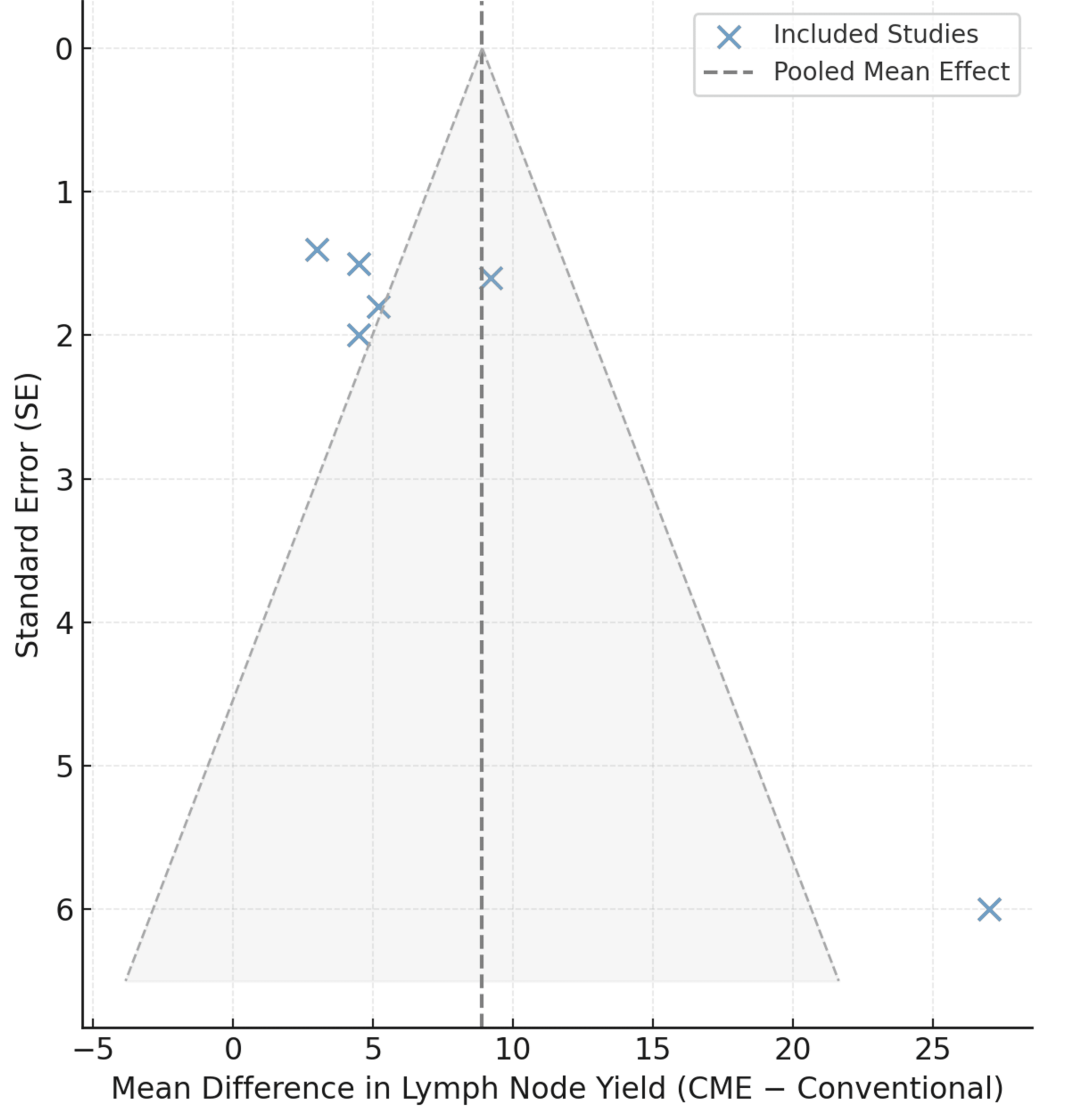
Funnel plot for publication bias assessment. Studies: Conti et al., 2021 [10]; Ouyang et al., 2019 [11]; Kadirvel et al., 2025 [12]; Mydin et al., 2024 [13]; Sztipits et al., 2023 [14]; Tümay et al., 2020 [15].

The funnel plot shown in Figure 3 illustrates the relationship between the effect sizes of the study, i.e., the standard difference in the lymph node yield of CME versus conventional colectomy, along with their standard errors. The dashed lines create a pyramid-shaped area, which is a 95% confidence limit of the pooled mean effect. All blue points are associated with one of the six observation studies. The overall symmetrical distribution about the central line of means indicates a low level of bias in publication. Smaller studies have higher standard errors that, when plotted, tend to be spread at the base, and large and more accurate studies tend to be concentrated at the top. Lack of significant asymmetry favors equal reporting and adds credibility to the aggregated meta-analytic results.

Discussion

This meta-analysis compared the results of CME against those of traditional right hemicolectomy on right-sided colon cancer patients. Six observational studies were included, and the results showed that CME can lead to an increase in lymph node yield and possibly better survival without perioperative morbidity or fatal consequences. The difference regarding operative time was small, although it appeared to be clinically acceptable. These results fill an important gap in knowledge about the effects of CME adoption on the short-term safety and quality of oncologic outcomes in common surgical practice.

The greater yield of lymph nodes seen in studies is consistent with previous meta-analyses due to evidence of the best oncologic clearance that has been realized by CME owing to thorough central vascular ligation [17]. The average difference in the pool of about six extra lymph nodes is probably an indication of the technical accuracy of the CME technique. Previous studies have shown that more harvested nodes give pathological staging more accuracy and can lead to better disease-free survival [18]. The results support these findings in that a more comprehensive mesocolic resection can strengthen disease control in the long term.

CME led to increased operation time, which would be expected as the dissection would go down to the central vascular root, and meticulous maintenance of the mesocolic plane needs to be maintained. However, this small rise did not lead to a rise in the morbidity rates. This trend represents the results of past European multicenter data, which showed that the incidences of complications were similar with CME and conventional colectomy [19]. In open and laparoscopic procedures, patient selection criteria and experience of the surgeon seem to have a strategic role in ensuring safe outcomes.

Even though the pooled survival analysis failed to meet the test of statistical significance, the direction of effects was in favor of CME concerning overall survival and disease-free survival. This can either mean a real oncologic benefit that was not adequately represented, given that small sample sizes and the nature of the data used were observational. Registry-based studies have also been found to indicate an improvement in survival in cases of CME over the long term, especially when conducted in high-volume centers where pathological audit is regularly conducted [20]. To that end, our results support the emerging future opinion that the quality of a surgical plane can directly influence the oncologic prognosis.

The low levels of heterogeneity seen among studies can be due to the differences in the expertise level of the surgeon or the pathology reporting procedures, as well as the diversity of patient groups. A few centers in Asia collected higher nodal yields due to a standardization of the handling of pathology, whereas the smaller centers in South Asia and Eastern Europe were found to have very poor averages [21]. Learning curves of CME and the variability in the definition of complete mesocolic dissection might have been one of the reasons behind the differences. The sensitivity analysis that picked out lower-quality or smaller studies slightly decreased the heterogeneity, thus validating the strength of the direction of the pooled findings [22].

This review is strong in its strict methodology. It fulfilled the requirements of PRISMA 2020 and MOOSE, used an extensive search strategy, and followed dual independent screening and data extraction. The NOS was carefully used to evaluate the risk of bias, whereas the Joanna Briggs Institute tool was used to check the quality. A random-effects model was used as a quantitative synthesis, which was suitable to explain the methodological and clinical variation. Funnel plots indicated that there was a low probability of publication bias.

Study limitations

Nevertheless, a number of limitations need to be mentioned. The selected studies were small, and they were all of the observational type. This has a narrow scope to cause an inference and the risk of unmeasured confounding. The hypothesis of laparoscopic versus open approach and technical specificity may have played a role concerning surgical heterogeneity. The definitions of CME and morbidity were also not similar, which would have implications for comparability. Furthermore, using English-language studies as the primary sources is likely to have led to language and regional bias, which could have omitted pertinent evidence in non-English-language journals. Duration of follow-ups was not fixed, and not all studies offered full survival information over a long-term period.

Despite these limitations, the results have clinical significance. The data suggest that CME is safe procedure to be conducted in a variety of surgical settings and can increase the pathological and even oncologic outcomes. These findings confirm the value of further training on mesocolic plane surgery as well as the need to use standard forms of operative procedure and pathology reporting techniques. The trends noted could be used to fund more research in the training of surgeons and an institutional audit of the quality of the mesocolic plane.

Where possible, future studies must incorporate larger and prospective observational cohorts or pragmatic randomized controlled studies. To establish survival effects and cost-benefit balance, long-term data are specifically required. The laparoscopic and robotic CME comparative studies could also aid in the improvement of minimally invasive techniques. In addition, international involvement in defining CME and methodological evaluation of pathology would enhance consistency of studies. The benefit of CME outcomes to populations could also be explained by integrating the results into national cancer registries.

## Conclusions

According to this systematic review and meta-analysis, CME in right-sided colon cancer is accompanied by increased lymph node harvest and a potential oncologic outcome enhancement with no added morbidity and mortality. The operative time was still slightly longer and was clinically acceptable. These results underline the oncologic and technical benefits of CME and prove its safety. Weaknesses consist of a small sample size, moderate heterogeneity, and the observational character of inclusive studies. Further national, interdisciplinary, and longitudinal studies are needed to validate these results and assess the cost and training implications of these findings in various healthcare settings.
